# Cucurbitacin B, Purified and Characterized From the Rhizome of *Corallocarpus epigaeus* Exhibits Anti-Melanoma Potential

**DOI:** 10.3389/fonc.2022.903832

**Published:** 2022-06-08

**Authors:** Sreekumar Usha Devi Aiswarya, Gowda Vikas, Nair Hariprasad Haritha, Vijayasteltar Belsamma Liju, Anwar Shabna, Mundanattu Swetha, Tennyson Prakash Rayginia, Chenicheri Kizhakkeveettil Keerthana, Lekshmi Raghu Nath, Mullan Vellandy Reshma, Sankar Sundaram, Nikhil Ponnoor Anto, Ravi Shankar Lankalapalli, Ruby John Anto, Smitha Vadakkeveettil Bava

**Affiliations:** ^1^Department of Biotechnology, University of Calicut, Malappuram, India; ^2^Division of Cancer Research, Rajiv Gandhi Centre for Biotechnology, Thiruvananthapuram, India; ^3^Chemical Sciences and Technology Division, Council for Scientific and Industrial Research (CSIR)-National Institute for Interdisciplinary Science and Technology (CSIR-NIIST), Thiruvananthapuram, India; ^4^The Shraga Segal Department of Microbiology-Immunology and Genetics, Faculty of Health Sciences, Ben-Gurion University of the Negev, Beer Sheva, Israel; ^5^Department of Pharmacognosy, Amritha School of Pharmacy, Amritha Vishwa Vidyapeetham, Amrita Institute of Medical Sciences (AIMS) Health Science Campus, Ponekkara P.O, Kochi, India; ^6^Agro-Processing and Technology Division, Council for Scientific and Industrial Research (CSIR)-National Institute for Interdisciplinary Science and Technology (CSIR-NIIST), Thiruvananthapuram, India; ^7^Academy of Scientific and Innovative Research (AcSIR), Ghaziabad, India; ^8^Department of Pathology, Government Medical College, Kottayam, India

**Keywords:** *Corallocarpus epigaeus*, Cucurbitacin B, melanoma, apoptosis, NMR spectroscopy, mass spectrometry

## Abstract

The ethnomedicinal plant from the Cucurbitaceae family, *Corallocarpus epigaeus*, or its bioactive derivatives have been widely utilized in traditional medicine owing to their distinct applications against various human ailments and have lured the interest of ethnobotanists and biochemists. Here, we report for the first time, the anti-cancer potential of a bio-active fraction isolated from the dried rhizome of *C. epigaeus*, and the bioactive principle identified as cucurbitacin B (Cu-B). The purification processes involving the utilization of multiple organic extracts of *C. epigaeus* rhizome powder, yielded Cu-B from the Ethyl acetate Cytotoxic Fraction (ECF), obtained by the chromatographic separation of the ethyl acetate extract. Amongst the various cancer lines tested, melanoma cells exhibit maximal sensitivity towards the Cu-B-containing ECF fraction. Cu-B induces an apoptotic mode of cell death initiated intrinsically as well as extrinsically in A375 melanoma cells whilst remaining comparatively less toxic to normal skin fibroblasts. *In vivo* studies involving a NOD-SCID murine model of human melanoma demonstrate the ability of Cu-B to attenuate tumor growth, while being pharmacologically safe *in vivo*, as assessed in *Swiss albino* mice. Furthermore, Cu-B inhibits MEK 1/2 as well as the constitutive and EGF-induced ERK 1/2 activation, indicating a definitive involvement of MAPK signal transducers in regulating Cu-B-mediated anti-melanoma activity. Together, our study demonstrates the anti-melanoma potential of *C. epigaeus*-derived Cu-B, which indicates the Cucurbitaceae succulent as a prospective source for deriving potent and pharmacologically safe anti-cancer compounds.

## Introduction

*Corallocarpus epigaeus* (Rottl. & Willd.) C. B. Clarke is a popular medicinal plant from the Cucurbitaceae family, notably prescribed in traditional medicine as a remedy for acute dysentery, venereal diseases, skin diseases, and snake bite (1). Despite its significance in traditional medicine, there are no comprehensive reports on the isolation of phytochemical constituents from *C. epigaeus*. Reports on the isolation of *N*-methyl asparagine from *C. epigaeus* seeds and essential oils like ishwarane and ishwarone from the roots are documented (2, 3). Volatile compounds from the roots and rhizomes of *C. epigaeus* have been detected by GC-MS analysis in comparison with standard phytochemical libraries for identification (3–5). In addition, there are reports on the identification of a pyridine carboxylic ester (corallocarpenoyl ester), an aliphatic C_32_ keto diol (dotriacont-22, 25-diol-10-one), a sesterpene lactone (corallocarpscalarolide), and a *p*-hydroxybenzoyl ester (designated as epigaeusyl ester) from the roots of *C.* epigaeus (6). Furthermore, a glycoside termed bryonin is purified from *C. epigaeus* roots (7). Organic solvent extracts of various parts of the plant are shown to possess a broad range of pharmacological properties (8). A couple of reports indicate that the ethanol extracts of *C. epigaeus* induce cytotoxicity in cancer cells (9, 10). However, a thorough investigation of the anti-cancer potential of *C. epigaeus* or its purified derivatives remains elusive.

In the present study, we report, the potent anti-cancer activity exhibited by the ethyl acetate extract of C*. epigaeus* dried rhizome and identify the anticancer principle in the extract as the triterpenoid, cucurbitacin B (Cu-B). Our studies demonstrate Cu-B as an efficacious agent against melanoma, compared to cancers of other tissue origins. Melanoma is the most deadly among skin cancer subtypes, the incidence, and mortality of which have been increasing over the past four decades (11). In melanoma, constitutive activation of MAP Kinase (MAPK) signaling *via* the RAS-B RAF -MEK-ERK signaling axis has been widely implicated in the initiation and development of cancer due to activation mutations of B-RAF and RAS genes. The development of small-molecule inhibitors of B-RAF, and MEK has made significant progress in melanoma chemotherapy. However, acquired resistance poses serious limitations to the therapeutic benefit of these small molecule inhibitors (12). A previous study has reported the ability of Cu-B to inhibit B-RAF and MEK by binding to the hydrophobic pocket of B-RAF receptor and allosteric site of MEK *via* molecular docking studies and has indicated the anti-melanoma potential of Cu-B by targeting the MAPK pathway (13). Our study indicates that Cu-B targets the MAPK pathway and evokes programmed cell death in melanoma cells by the induction of the apoptotic machinery. The pharmacological safety of Cu-B is ensured by its treatment on the normal skin fibroblasts. *In vivo* xenograft and toxicological studies corroborated the anti-melanoma efficacy and pharmacological safety of Cu-B. Taken together, our study reports the isolation and characterization of Cu-B from *C. epigaeus* rhizome and its prospective anti-melanoma potential.

## Materials and Methods

### Reagents and Antibodies

Cell culture reagents such as Dulbecco’s Modified Eagle Medium (DMEM) (GIBCO, 12800-017) and streptomycin sulfate (GIBCO, 11860-038) were obtained from Invitrogen Corporation (Grand Island, USA). Poly Excel HRP/DAB detection system universal kit (PathnSitu Biotechnologies Pvt. Ltd, India, OSH001) was used for immunohistochemistry experiments. Cucurbitacin B and MTT reagent were purchased from TCI Chemicals (India) Pvt. Ltd (D0801) and Amersham ECL Plus™ Western blotting reagents (PRPN 2132) were purchased from GE Healthcare Life Sciences (Piscataway, USA). Annexin V apoptosis detection kit (sc4252AK) was purchased from Santa Cruz Biotechnology (Santa Cruz, CA, USA). Antibodies against, Caspase 9 (9508S), Caspase 8 (4790S), Caspase 7 (12827S), Bid (2002S), p-P53 (9281S), PARP (9532S), p-ERK1/2 (4370S), ERK (9108S), p-STAT3 (9136S), β-actin (12620S) and p-MEK1/2 (9121S) were obtained from Cell Signalling Technologies (Beverly, MA, USA) and the antibody against C-MYC (sc764), Cyclin-D1 (sc8396), PCNA (sc25280) were purchased from Santa Cruz Biotechnology (Santa Cruz, CA, USA).Antibody against Caspase 3 (74T2) and ECL reagent (Pierce™ ECL western blotting substrate 32109) were purchased from ThermoFisher Scientific (Waltham, Massachusetts, United States) Antibody against MITF-M (ab12039) and Cellular ROS kit (ab113851) were purchased from Abcam (Cambridge, United Kingdom). Anti-caspase 10 (BD 51-9000066) antibody was purchased from BD Bioscience. DeadEnd™ Colorimetric TUNEL System from Promega (G7132) was procured from Addgene (Cambridge, MA, USA). An antibody against B-RAF^V600E^ (SAB 5600047), anti-rabbit antibody, anti-mouse antibody, and silica gel for column chromatography were obtained from Sigma Chemicals (St. Louis, MO, USA). EGF was purchased from Genscript (New Jersey, U.S). Organic solvents and TLC sheets were purchased from Merck (Germany). All other chemicals and an antibody against Vinculin (V9131) were purchased from Sigma Chemicals (St. Louis, MO, USA) unless otherwise mentioned.

### Cell Lines

The lung cancer cell line, H1299, and normal skin fibroblast, FS were gifts from Prof. B.B. Agarwal to RJA. The cancer cell lines viz. colon (HCT-116), breast (MDA-MB-231), liver (HEP 3B), and cervical (HeLa) were procured from NCCS, Pune, India. Melanoma cell lines with different mutation status viz. A375 (B-RAF), SK-MEL-2 (N-RAS), and SK-MEL-28 (B-RAF) were procured from NCCS, Pune, India. All the cells were routinely maintained in a complete medium, which contained DMEM, 10% FBS, and 2mg/ml Amphotericin B. The cells were incubated at 37°C and 5% CO_2_ atmosphere. Mycoplasma tests were performed on parent cell lines every 6 months. Cell lines passage between 3-6 times post-revival, were used for all experiments.

### Plant Specimens

Fresh Rhizomes of *C. epigaeus* collected in January 2017 from Nagamalai, Madhurai were identified and authenticated by Dr. Pradeep Kumar, Curator, Department of Botany, University of Calicut, and a voucher specimen has been deposited at the Department of Botany, University of Calicut (VOUCHER NO: CALI 6891).

### Preparation of Extracts and Isolation of ECF

Hexane, ethyl acetate, and methanol extracts were prepared by polarity gradient successive extraction of the dried rhizome powder. Among the three organic extracts of the rhizome, ethyl acetate extract was found to be the most cytotoxic. To isolate the active principle, we subjected the ethyl acetate extract to column fractionation, which yielded 11 fractions. Chromatographic separations were carried out by conventional column chromatography on silica gel (100-200 and 230-400 mesh).We tested the cytotoxic activity of each fraction among which, four fractions (Fraction 6 to 9) were found to be highly cytotoxic. As they showed similar cytotoxic profile as well as TLC pattern, we pooled them together and designated them as ECF (Ethyl acetate Cytotoxic Fraction).

### Nuclear Magnetic Resonance Spectroscopic Analysis

^1^H and ^13^C NMR were recorded on a Bruker ASCENDTM-500 spectrometer at 500 and 125 MHz, respectively using CDC_l3_ and acetone-d6 solvents. TOCSY spectrum was acquired with an 80 ms mix time. NMR data are reported as follows: chemical shifts in ppm (δ) with integration, coupling constants in Hz. ^1^H, ^13^C, and 2D NMR data were used to elucidate the structure of the compounds.

### Mass Spectrometry

Higher Resolution Mass Spectrometry (HRMS) analysis was recorded to determine the molecular formula of the compounds using a Thermo Scientific Exactive-Liquid Chromatography-Mass Spectrometry (LCMS) instrument by electrospray ionization method with ions given in *m/z* using Orbitrap analyzer.

### Ultra-High Performance Liquid Chromatography Profiling of ECF Extract From *C. epigaeus*

The sample was injected into the analytical Nexera UHPLC system equipped with a reverse-phase Shim-pack GWS 5µ C18 column 250 × 4.6 mm ID connected to a PDA detector (SPD-M20A) and an autosampler (SIL-30AC). ECF fraction (3 mg/ml) and the isolated pure compound (2 mg/ml) were dissolved in acetonitrile: water (1:1) and filtered through a 0.2 µm nylon filter. The sample injection volume was 20 μL, and the C18 column temperature was 35°C. The mobile phase system consisted of water: acetic acid (100:1) (A) and acetonitrile (B). A step gradient program was used for this analysis as follows: 0% B at 0 min to 40% B at 20 min, 40 to 50% at 30 min, 50 to 60% at 40 min, 60 to 80% at 50 min, 80 to 100% at 60 min, then maintaining at 100% B from 60 to 65 min at a flow rate of 1 ml/min, monitored at 254 nm.

### MTT Assay

The cells were seeded in 96-well plates (2000 cells/well), incubated overnight, and treated with different concentrations of plant extracts, ECF and Cu-B. After 72 h the sample solution was flicked off and Fresh media containing 25 µL of 3-(4, 5-Dimethylthiazol-2-yl)-2,5-Diphenyltetrazolium Bromide (MTT) solution (5 mg/mL in PBS) was added to the wells and incubated for 2h. At the end of incubation, lysis buffer (20% sodium dodecyl sulfate in 50% dimethylformamide) was added to the wells (0.1 mL/well) and incubated for another 1 h at 37°C. At the end of incubation, the optical density was measured at 570 nm using an ELISA plate reader (Bio-Rad). The relative cell viability in percentage was calculated as (A570 of treated cells/A570 of untreated cells) X 100. The IC50 values were extrapolated from polynomial regression analysis of experimental data.

### Clonogenic Assay

Clonogenic assay or colony formation assay is an *in vitro* cell survival assay based on the ability of a single cell to grow into a colony. The colony is defined to consist of at least 50 cells. The assay essentially tests every cell in the population for its ability to undergo “unlimited” division. Briefly, 500 cells were seeded in 6-well plates and treated with different concentrations of ECF. After 72 h, media along with ECF was removed, supplied with fresh medium, and incubated for 1 week. The developed clones were fixed in glutaraldehyde (6%) and stained using crystal violet (0.5%). The plate was incubated for 30 min at room temperature, followed by rinsing with tap water. After drying the plate, colonies were counted and compared with the untreated control.

### Annexin V-Propidium Iodide Staining

Apoptotic cells were detected with the help of a fluorescent microscope by Annexin V apoptosis detection kit according to the manufacturer’s protocol (Santa Cruz, CA, USA). Briefly, the cells were seeded in 96-well plates and treated with ECF as in the MTT assay, but for 16 h. The cells were then washed with PBS, followed by 1X assay buffer, after which, 5 µL of Annexin V FITC and 10 µL of propidium iodide per 100 µL assay buffer was added, followed by incubation in the dark for 15min. The cells were then washed with PBS and immediately photographed using a fluorescent microscope, Nikon inverted fluorescent microscope (TEEclipse 300).

### Fluorescent Activated Cell Sorting Analysis for Apoptosis

The extent of apoptosis induced by ECF and Cu-B was estimated by FACS using Annexin V FITC apoptosis kit (Santa Cruz, CA, USA). Briefly, the cells were seeded in 60mm culture plates, and incubated with different concentrations of ECF and Cu-B. After 16 h, cells were trypsinized and the pellets were washed with PBS and suspended in 100 µL 1 X assay buffer. To the buffer, 5 µL of FITC conjugated Annexin V and 10 µL of propidium iodide were added and incubated for 15 min in dark at room temperature. The cells were then analyzed by flow cytometry to get the percentage of apoptotic cells (FACS Aria™, BD Bioscience)

### Flow Cytometry and Cell Cycle Analysis

Cell cycle analysis helps in differentiating the distribution of a population of cells to the different stages of the cycle. Briefly, cells were treated with different concentrations of Cu-B and incubated for different time periods (24 h and 48 h). After incubation, the cells were trypsinized and the pellets were washed with PBS, and fixed in 70% ice-cold ethanol treated with 100 mg/ml RNAase A and 50 mg/ml propidium iodide, followed by flow cytometric analysis (BD Biosciences).

### Fluorescent Microscopy for Reactive Oxygen Species

ROS levels within the cells in response to Cu-B were determined by staining the cells using H2DCF-DA according to the manufacturer’s protocol. Briefly, the cells were seeded in 60 mm plates, kept overnight, and treated with different concentrations of Cu-B for 6 h followed by trypsinization. The cell pellets were washed with PBS, re-suspended in DCFDA containing assay buffer, and incubated for 30 min. After incubation, the cells were washed with PBS and observed and quantified using a Nikon inverted fluorescent microscope (TEEclipse 300).

### Immunoblot Analysis

The cells were treated with the indicated concentration of ECF/Cu-B. The whole-cell lysates of drug treated cells/Tissue extracts from Cu-B treated mice, were electrophoresed by SDS/PAGE, and electrotransferred to PVDF membranes, the membranes were blocked with 5% milk for 60 min, washed using TBST and immunoblotted with the appropriate antibodies (14). The bands were visualized using an enhanced chemiluminescence kit (Pierce™ ECL western blotting solution) as per the manufacturer’s protocol.

### Animal Experiments

#### Toxicological Analyses

The toxicological analysis of the active fraction ECF and Cu-B were performed in 6-8 weeks old male *Swiss albino* mice as per protocol (IAEC/669/RUBY/2018 and IAEC/849/Ruby/2021) approved by the Institutional Animal Ethics Committee, Rajiv Gandhi Centre for Biotechnology.

*Acute toxicity:* Doses of 0, 0.25, and 1.25mg/Kg of active fraction ECF and 0, 0.05, and 0.15 mg/Kg of Cu-B were given as a single intraperitoncinsiseal injection to each group of 5 animals. The mice were observed continuously for 1 h, for any gross behavioral changes and death, and then intermittently for the next 6 h and 24 h. The behavioral parameters monitored were convulsion, hyperactivity, sedation, grooming, food and water intake, etc. The animals were observed routinely for the next 7 days from the day of treatment, after which, the animals were euthanized. The liver tissue was verified by histopathologic evaluation using H&E staining and the serum was used to perform Liver Function Test (14).

*Sub-chronic toxicity:* Doses of 0, 0.25, and 1.25mg/Kg of active fraction ECF and 0, 0.05, and 0.15 mg/Kg of Cu-B were given as intraperitoneal injections on alternate days for 3 months. Each group consists of 5 animals. Animals were euthanized after 3 months. Liver tissues were collected and toxicity was measured as described above.

#### *In Vivo* Xenograft Model

A melanoma xenograft study was performed in male NOD-SCID mice following the approved guidelines of the Institute Animal Ethics Committee of Rajiv Gandhi Centre for Biotechnology, Thiruvananthapuram (IAEC/818/RUBY/2020). A375 cells (1X10^6^) were subcutaneously injected into the flank region of the mice. 5 days post the cell injection, the animals were divided into 3 groups. Cu-B being hydrophobic was encapsulated in the liposomal formulation, which was prepared by vacuum rotary evaporation of a mixture of 1 mg Cu-B, 9 mg phosphatidylcholine, and 1.16 mg cholesterol, dissolved in a 3:1 mixture of chloroform and methanol. 0.05 mg/Kg of liposomal Cu-B (corresponding to the IC50 in A375), was prepared by dissolving the liposome in 1X PBS and administered intradermally and intraperitoneally to the animals in groups 2 and 3 respectively, on alternative days for a period of 4 weeks. Group 1 was kept as vehicle control. The tumor size was measured weekly and the corresponding tumor volume was calculated as per the formula, (length x width^2^)/2 (15). At the end of the experiment, the animals were euthanized and tumor tissues were collected.

#### Histology and Immunohistochemistry

The tumor and liver tissues from mice were fixed and sectioned and stained using Hematoxylin and eosin (H & E). Immunolocalization of specific proteins in the tissue sections was done using Poly Excel HRP/DAB detection system universal kit for mouse and rabbit primary antibodies as per the manufacturer’s protocol. The tissues were subjected to an immunohistochemical analysis against, PCNA (Proliferating Cell Nuclear Antigen), B-RAF^V600E^, p-MEK1/2, p-ERK1/2, p-STAT3, c-MYC, Cyclin-D1 and β-Catenin. All the immunohistochemistry images were taken in DMi8 Inverted Fluorescence Research Microscope with a DMC 2900 Digital Camera.

#### TUNEL Assay

TUNEL assay was performed to detect apoptosis in formalin-fixed, paraffin-embedded xenograft tumor tissue sections using Dead End Colorimetric TUNEL System (Promega) following the manufacturer’s instructions.

### Statistical Analysis

For the flow cytometry, data analysis was performed using the BD FACS Diva software version 5.0.2. The statistical analysis was performed using Graph Pad Prism software Inc. (version6.0, San Diego, CA, USA) and the quantification of Western blot was carried out using ImageJ software, version1.8.0. Statistical significance was defined as p < 0.05. The error bars represent SD, taken from three independent experiments.

## Results

### ECF, a Bioactive Fraction From *C. epigaeus* Rhizome Exhibits Anti-Melanoma Activity

**Figure 1:**
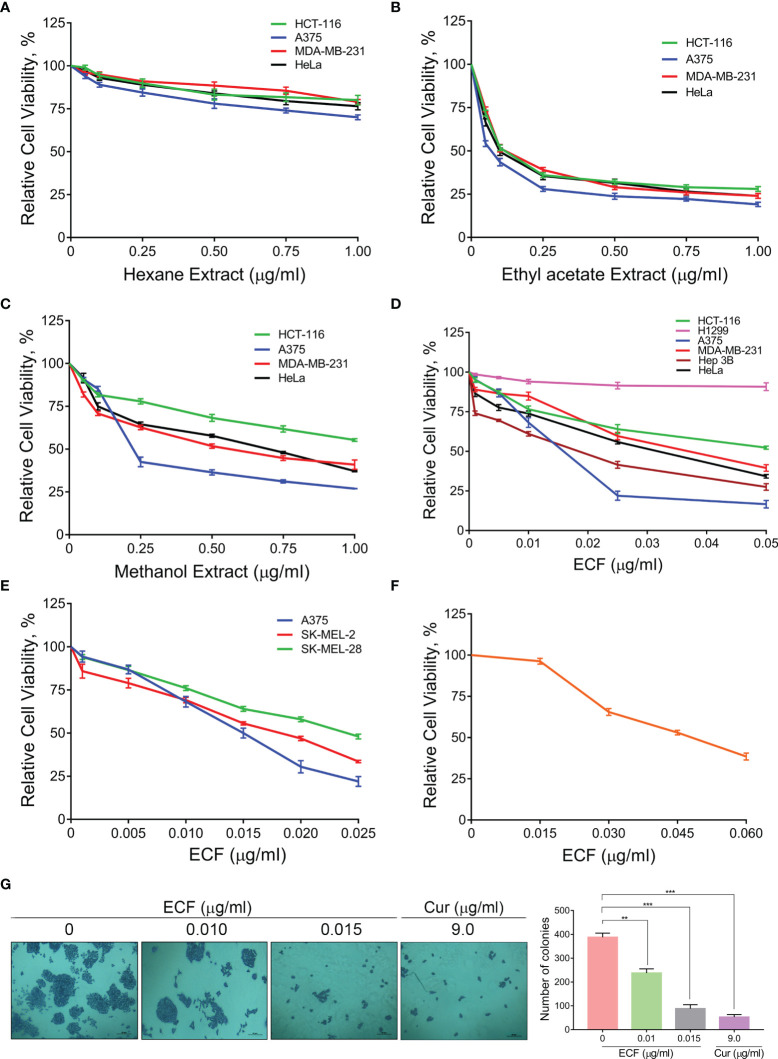
ECF, a bioactive fraction of *C. epigaeus* rhizome induces cytotoxicity in melanoma cells **(A–C)** Cytotoxicity assessment of the *C. epigaeus* rhizome-derived organic extracts on cancer cells of various tissue origins. **(D)** Cell viability analysis upon treatment by ECF bioactive fraction as assessed in various cancer cell lines. **(E, F)** Cytotoxicity induced by ECF on melanoma cell lines in comparison to normal skin fibroblast, FS cells. **(G)** ECF inhibits the proliferative potential of A375 cells. Data are representative of three independent experiments (Mean±SEM) and P-values are calculated using one way ANOVA. ***P ≤0.001, **P ≤0.01 and ns ≥ 0.05.

To investigate the potential anti-cancer ability of *C. epigaeus*, the rhizome part was preferred, due to its multiple applications in traditional medicine against various ailments. A polarity gradient extraction of *C. epigaeus* dried rhizome powder was performed and the cytotoxicity analysis using the three organic extracts (hexane, ethyl acetate, and methanol) was conducted in cancer cell lines of various tissue origins. The assessment of cell viability in the indicated cancer cells demonstrated the ethyl acetate extract as the most potent fraction which exhibited substantial cytotoxicity with an IC50 around 0.05µg/ml, particularly in the melanoma cell line, A375. The methanol extract also possessed the ability to induce death, most notably in A375 (IC50 of 0.15 µg/ml) whereas the hexane fraction was found to be non-toxic to all cell lines (**Figures 1A–C**). The ethyl acetate extract was considered for further studies to isolate and purify one or more potential anticancer principle(s). We subjected the ethyl acetate extract to silica gel column chromatography, which yielded a bioactive fraction, and was designated as “ECF” [the isolation of ECF has been detailed in the methodology section]. We tested the cytotoxic ability of ECF against various human cancer cell lines using a cell viability assay. The melanoma cell line, A375 displayed maximal sensitivity towards ECF (IC50- 0.015 µg/ml) (**Figure 1D**). To validate the anti-melanoma efficacy of ECF, we tested it on different melanoma cell lines viz. A375, SK-MEL-2, and SK-MEL-28. Interestingly, all the melanoma cell lines selected for this study displayed considerable sensitivity towards ECF. The cell line, A375 was repeatedly observed as the most sensitive to ECF and was picked for further studies (**Figure 1E**). Prior to the studies in A375, we tested the biological safety of the ECF, by treating normal skin fibroblast cells, FS, and found that the IC50 concentration of ECF in these cells was three times higher than that in A375 cells (**Figure 1F**). We further studied the ability of ECF to inhibit the proliferative propensity of A375 cells using a clonogenic assay. The widely studied bio-active phytochemical curcumin (9 µg/ml), was used as a positive control. The result indicated a significant reduction in the number and size of the colonies formed, demonstrating the anti-melanoma potential of ECF (**Figure 1G**). The results suggest *C. epigaeus*-derived ECF as a highly efficacious bioactive fraction potent to kill melanoma cells.

### ECF Induces Caspase-Dependent Apoptosis in Melanoma Cells

**Figure 2:**
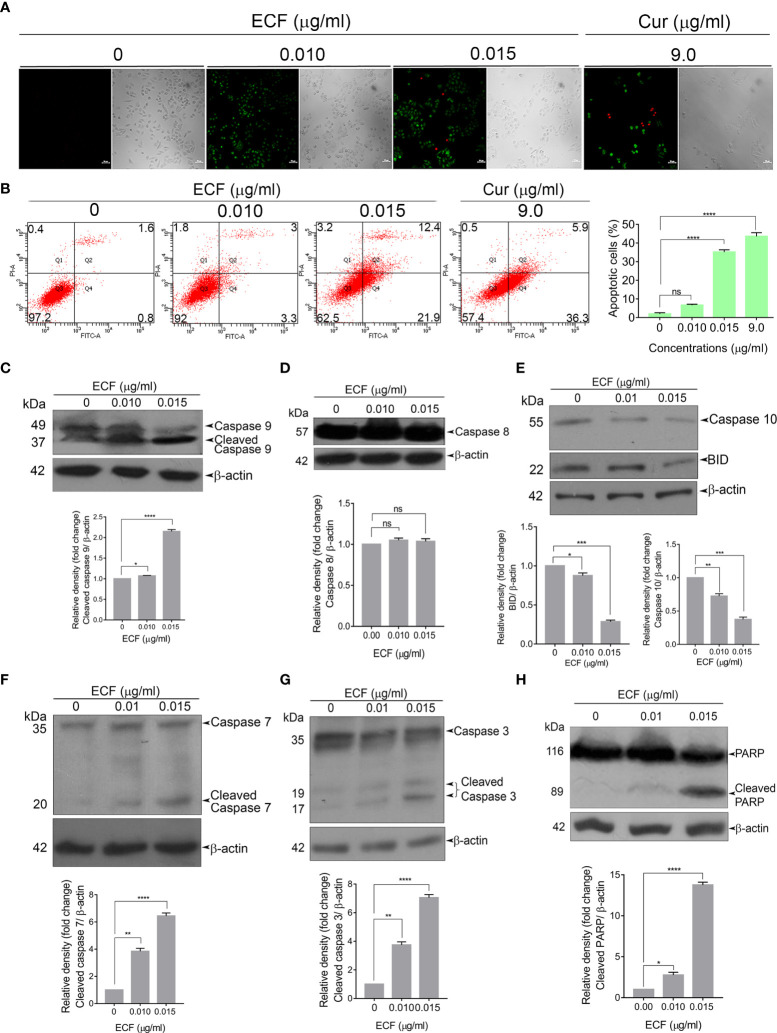
ECF triggers apoptotic mode of cell death in melanoma **(A, B)** ECF induces apoptosis in A375 cells as assessed by Annexin/PI staining, and was quantitated by FACS analysis. **(C–H)** ECF potentiates the activation of caspases and cleavage of PARP in A375 cells as analyzed by immunoblotting. Data are representative of three independent experiments (Mean±SEM) and P-values are calculated using one way ANOVA. ****P ≤0.0001, ***P ≤0.001, **P ≤0.01, *P ≤0.1and ns ≥ 0.05.

Next, to analyze the mode of cell death induced by ECF in melanoma, we tested the efficacy of ECF to induce apoptosis using FITC-Annexin V/PI staining. ECF treatment led to a significant hike in the number of FITC/PI+ apoptotic cells in comparison to the untreated control (**Figure 2A**). The extent of apoptosis induced by ECF was further estimated by FACS analysis of the Annexin V–FITC/PI double-stained cells. The apoptotic cell population was increased from 2.4% to 6.3% and 34.3% respectively when treated for 16 h with 0.01 μg/ml and 0.015 μg/ml of ECF. A375 cells treated with curcumin (9 µg/ml) were used as a positive control (**Figure 2B**). We further tested the cleavage of procaspases, a marker of the apoptosis program, using immunoblotting. Apoptosis is mediated by cysteine proteases termed caspases, which are functionally classified into initiators and executioners. Initiator caspases 8 and 9, activated by intrinsic or extrinsic apoptotic stimuli, subsequently activate executioner caspases, which in turn cleave the cellular death substrates, eventually resulting in apoptosis. ECF treatment led to the cleavage of the initiator caspase 9 (**Figure 2C**) while caspase 8, associated with extrinsic stimuli, remained unaffected (**Figure 2D**). Further, we tested the activation of BID, a specific proximal substrate of caspase 8 in the death receptor-mediated extrinsic apoptotic signaling pathway and a mediator of caspase 8-induced mitochondrial damage (16). Strikingly, we noticed an activation of BID upon ECF treatment, even in the absence of caspase 8 activation (**Figure 2E**). Previous reports indicate the ability of initiator caspase 10 to cleave and activate BID (17). Indeed, we observed a significant reduction in the procaspases 10 level indicating caspase 10 activation (**Figure 2E**) by the fraction. Furthermore, ECF induced the cleavage of executioner caspases, 7and 3, as well as the substrate of executioner caspases, Poly ADP-Ribose Polymerase (PARP) (**Figures 2F–H**). Together, these data confirm that the cytotoxicity induced by ECF in melanoma cells involves caspase-dependent apoptosis.

### ECF is Pharmacologically Safe, *In Vivo*

To ascertain the biological safety of ECF, we conducted acute and sub-chronic toxicity studies in *Swiss albino* mice. For this, we did a mass spectrometry analysis of the ECF fraction and calculated the optimal dose using the Castanas method, taking the molecular weight corresponding to the base peak obtained as 581, which corresponds to a sodium adduct of 558, assuming that this compound is responsible for the cytotoxic effect of the fraction (18). We selected two doses of ECF, the optimal dose and five times the optimal dose [0.25 and 1.25 mg/kg], for the drug-induced toxicity evaluation. The murine blood was collected to quantitate AST, ALT, and ALP, the elevated levels of which are indicative of liver toxicity. Histopathological analysis of mice liver tissues was also performed using H & E staining. Notably, no behavioral changes such as convulsion, hyperactivity, sedation, grooming, food and water intake, etc. were observed in the mice upon ECF treatment. In addition, no significant changes were observed in the body weight of the animals. The serum analysis, as well as histopathological evaluation of liver sections of the mice studied, did not show a significant toxicological change in any of the parameters studied (**Figures S1A–F**), indicating that ECF is pharmacologically safe *in vivo*.

### The Triterpenoid, Cucurbitacin B, Is the Anticancer Principle in ECF

**Figure 3:**
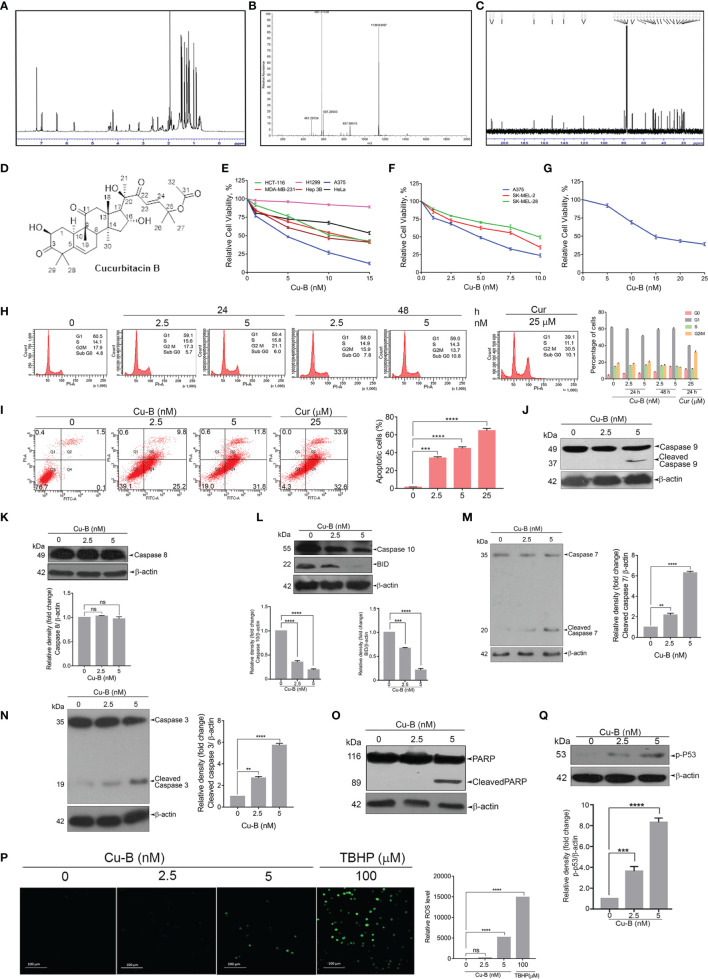
Purification and structural elucidation of Cu-B from ECF fraction of *C. epigaeus* rhizome **(A–D)** ^1^H NMR, HR-ESI-MS, ^13^C NMR data, and structure of *C. epigaeus*-derived Cu-B. **(E–G)** Cytotoxicity assessment of Cu-B in melanoma cell lines and normal fibroblast cells **(H)** Cu-B does not affect any phases of the cell cycle in A375 cells as demonstrated by flow cytometry. **(I)** The extent of apoptosis induced by Cu-B was quantitated by Annexin V/PI FACS analysis **(J–O)** Cu-B treatment induces activation of caspases and cleavage of PARP in A375 cells as analyzed by immunoblotting. Cu-B promotes the cleavages of caspase-10 and Bid as analyzed by immunoblotting. **(P)** ROS production in response to Cu-B treatment in A375 cells as detected by fluorescence microscopy. **(Q)** Cu-B potentiates p53 activation in A375 cells as analyzed by immunoblotting. Data are representative of three independent experiments (Mean±SEM) and P-values are calculated using one-way ANOVA. ****P ≤0.0001, ***P ≤0.001, **P ≤0.01 and ns ≥ 0.05.

We further focused on the isolation, purification, and identification of the anticancer compound in ECF. Column chromatography of ECF led to a major product with polar characteristics. An initial observation by ^1^H NMR indicated a triterpene pattern with polar functional groups for the isolated compound (**Figure 3A**). The compound was found to have structural similarities with cucurbitacins, which are abundantly oxygenated triterpenes. Initially, an HR-ESI-MS analysis was carried out to match the list of already isolated cucurbitacins from the family of Cucurbitaceae. The molecular formula of the *C. epigaeus*-derived cucurbitacin was determined as C_32_H_46_O_8_ as per the HR-ESI-MS analysis which exhibited ions at 581.3113 (M+Na)+ along with a corresponding dimer peak at 1139.6336 (2M+Na)+ (**Figure 3B**). The ^13^C NMR exhibited three ketone functional groups at δC 202.50, 212.19, and 213.08 ppm, and one among these three carbonyls is a part of α, β-unsaturated system that appears at δH 6.4 (d, J = 16.5 Hz) and 7.0 (d, J = 16 Hz) in the ^1^H NMR (**Figure 3C**). The presence of δC 170.32 indicated the presence of an ester group and the presence of nine methyl groups in ^13^C NMR. Together, the spectral data indicated the identity of the polar compound as cucurbitacin B (purity 99.99%) (**Figure 3D**). Gratifyingly, the ^1^H and ^13^C NMR were found to be in perfect agreement with published literature (**Table S1**) for Cu-B (19). This is the first report indicating the presence of Cu-B from *C. epigaeus*. UHPLC profiling was conducted to ascertain the purity of the isolated Cu-B, which exhibited a peak at 31.8 min at 254 nm. The chromatograms of Cu-B and its presence in ECF were confirmed by spiking the bioactive fraction with pure cucurbitacin B (**Figures S2A, B**).

### Cucurbitacin B Possesses Potent Anti-Melanoma Activity

Owing to the potent anticancer activity of ECF against melanoma cell lines, we sought to explore the anti-melanoma efficacy of Cu-B *in vitro*. In line with the observation in ECF fraction (refer to **Figure 1D**) we observed increased sensitivity of the melanoma cells to Cu-B in comparison to cancer cells of other tissue origins (**Figure 3E**). Melanoma is classified into several molecular subgroups based on genomic alterations, among which B-RAF and NRAS mutated melanomas are the most common (20). Therefore, we selected melanoma cell lines, which belong to the two molecular subgroups, with B-RAF/NRAS mutation status viz. A375 cells [B-RAF mutation], SK-MEL-2 [NRAS mutation] and SK-MEL-28 [B-RAF mutation]. Cu-B induced potent cytotoxicity in all the three cell lines chosen for the study, irrespective of the mutation status (**Figure 3F**). Again A375 cell line, which exhibited maximal sensitivity to Cu-B (IC50-5nM), was selected for further studies. To confirm the biological safety, we tested the cytotoxic effect of Cu-B on normal skin fibroblasts and the IC50 concentration was found to be thrice that observed in the melanoma cell line, A375 (**Figure 3G**).

To delineate the mode of cell death induced by Cu-B in A375 cells, we analyzed whether Cu-B induces any cell cycle-specific effects by flow cytometry, and it was found that Cu-B did not interfere with any phases of the cell cycle even after prolonged treatment for 48 h (**Figure 3H**). Notably, there was an augmentation in the number of cells at the sub G0 phase, which was an indication of apoptosis. To quantitate the extent of apoptosis induced by Cu-B, we conducted FACS analysis of the Cu-B treated, Annexin V–FITC/PI double-stained cells. It was interesting to see that the apoptotic cell population increased from 1.6% to 35% and 43.6% respectivelywhen treated with 2.5 nM and 5 nM of Cu-B for 16 h. A375 cells treated with 25 µM curcumin were used as positive control (**Figure 3I**). To ascertain the Cu-B-mediated apoptotic mode of cell death in melanoma, we performed immunoblot analysis focusing on cleaved activated caspases and PARP, which are effective markers of apoptosis. We found a dose-dependent cleavage of the initiator caspase 9 as well as the effector caspases, 3 and, 7 (**Figures 3J, M, N**). Interestingly, Cu-B failed to induce the cleavage of caspase 8, as observed upon ECF treatment (**Figure 3K**), and in line with the data obtained with ECF, we observed activation of caspase 10 and BID in A375 cells indicating the involvement of the death receptor pathway in Cu-B induced apoptosis (**Figure 3L**). Furthermore, Cu-B triggered a noticeable cleavage of PARP, marking apoptotic cell death (**Figure 3O**). Previous reports have demonstrated the ability of chemotherapeutic drugs to induce apoptosis in cancer cells by triggering DNA damage as a result of increased ROS production (21). To investigate whether Cu-B elevates ROS production in A375 cells, a ROS-sensitive H2DCF-DA assay was employed. Cu-B treatment in A375cells triggered the oxidation of H2DCF-DA by ROS to dichlorofluorescein (DCF), which further led to the generation of green fluorescence, the intensity of which was quantified by confocal microscopy (**Figure 3P**). Moreover, we observed an increase in the phosphorylation of p53 in response to Cu-B (**Figure 3Q**). These results indicate that Cu-B augments ROS production in A375 cells, subsequently leading to intrinsic apoptosis, signaled by DNA damage-induced p53 signaling. Together, our data demonstrate the cell death mechanism through which *C. epigaeus*-derived Cu-B targets melanoma cells with the involvement of extrinsic and intrinsic apoptotic pathways.

### Cucurbitacin B Targets MAPK Signaling in Melanoma

**Figure 4:**
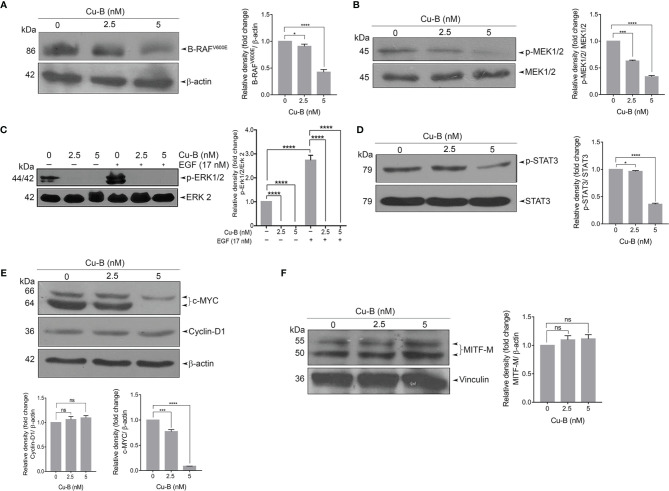
The effect of Cu-B on the key survival signals in melanoma **(A, B)** Cu-B diminishes B- RAF^V600E^ and p-MEK1/2 expressions in A375 cells. **(C)** Cu-B down-regulates the constitutive and EGF-induced phosphorylation of ERK1/2 in A375 cells. **(D, E)** Cu-B reduces the expressions of p-STAT 3 and c-MYC, but not Cyclin-D1, in A375 cells. **(F)** Cu-B treatment unalters MITF-M levels in A375 cells. Data are representative of three independent experiments (Mean±SEM) and P-values are calculated using one-way ANOVA. ****P ≤0.0001, ***P ≤0.001, *P ≤0.1and ns ≥ 0.05.

The constitutive activation of RAS-RAF-MEK-ERK signaling axes has been widely implicated in the initiation and development of melanoma *via* the activation of mutant RAF and RAS proteins (22). We tested the effect of Cu-B on MAPK signaling firstly by analyzing the expression status of B-RAF protein in A375 cells, which endogenously harbor a B-RAF mutation. The result revealed a significant down-regulation of the mutant B-RAF^V600E^ protein (**Figure 4A**). Further, we looked for the activation of MEK and ERK by immunoblot analysis of MEK1/2 and ERK1/2 phosphorylation. Notably, Cu-B treatment ablated the constitutive phosphorylation of MEK1/2 and ERK1/2 (**Figures 4B, C**), indicating Cu-B-mediated suppression of B-RAFV600E downstream kinase activity. We further analyzed the status of phospho-STAT3, a major transcription factor that stays downstream of the RAF pathway and is involved in maintaining cell proliferation and survival in melanoma (23). Active ERK is reported to phosphorylate Ser^727^ residue of STAT3 (24). We observed a significant down-regulation in the levels of phospho-STAT3 upon Cu-B treatment of A375 cells (**Figure 4D**). We also tested the effect of Cu-B on the expression statuses of c-MYC, and Cyclin-D1, the major downstream targets in the MAPK pathway. Notably, the Cyclin-D1 protein levels remained unaltered, however, a down-regulation in the c-MYC levels was observed (**Figure 4E**). In addition, we analyzed the expression levels of MITF-M, and β-catenin in A375 cells, however, Cu-B treatment did not affect their statuses in melanoma (**Figure 4F** and **Figure S4A**). The serum analysis, as well as histopathological evaluation of liver sections from Cu-B-treated mice did not reveal any noticeable toxicological changes in any of the parameters tested (**Figures S3A-F**), indicating the pharmacological safety of Cu-B. Collectively, our data demonstrate the key factors which are targeted by Cu-B to exert its potency against melanoma.

### Cucurbitacin B Suppresses Melanoma Growth in a NOD-SCID Tumor Model

**Figure 5:**
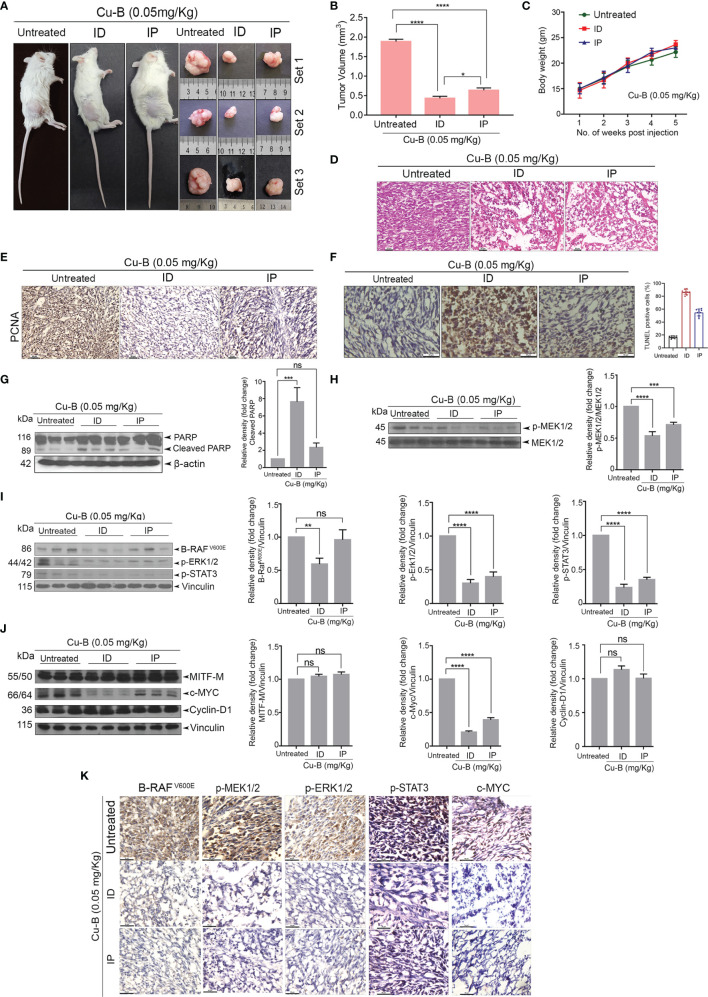
Cu-B suppresses tumor development in a NOD-SCID murine model of human melanoma **(A)**. Representative photographs of Cu-B treated NOD-SCID mice bearing A375 xenografts and the excised tumors. **(B)** Cu-B represses the tumor volume in Cu-B treated melanoma-bearing mice. **(C)** Cu-B treatment unalters the bodyweights of mice throughout the study period. **(D)** Histopathological evaluation of tumor tissue isolated from mice groups. Formalin-fixed cryosections were H and E stained. **(E)** IHC analysis on the expression of PCNA in tumor tissues of mice groups**. (F)** The apoptosis induced by Cu-B in the tumor tissues was confirmed by TUNEL assay **(G)** Immunoblot analysis demonstrating enhanced cleavage of PARP in the tumor lysates of Cu-B treated mice. **(H–J)** Immunoblot analysis showing the effect of Cu-B on key survival signals in melanoma, B- RAF^V600E^, p-MEK1/2, p-ERK1/2, p-STAT3, c-MYC, Cyclin-D1, and MITF-M, as evidenced in the tumor lysates. **(K)** IHC analysis on the expression of B- RAF^V600E^, p-MEK1/2, p-ERK1/2, p-STAT3, and c-MYC in tumor tissues of mice groups. Data are representative of three independent experiments (Mean±SEM) and P-values are calculated using one-way ANOVA. ****P ≤0.0001, ***P ≤0.001, **P ≤0.01, *P ≤0.1 and ns ≥ 0.05.

The anti-melanoma efficacy of Cu-B was tested *in vivo* using a xenograft model of human melanoma in NOD-SCID mice using A375 cells. The development of the A375-induced tumor and drug treatment regimen in the NOD-SCID murine model has been detailed in the methodology. Following the regimen, the tumors from the animals were excised for further analysis. Firstly, we observed that the ID administration of Cu-B was more effective, as evidenced by the significant reduction in tumor volume, compared to that of IP drug administration (**Figures 5A, B**). The body weight of the animals was routinely checked and no significant change between the groups was observed (**Figure 5C**). Histopathological analysis indicated substantial destruction of tumor cells in Cu-B-treated mice-derived tissues, which correlated with the considerable tumor reduction (**Figure 5D**). Immunohistochemical analysis for the expression of PCNA in tumor sections derived from Cu-B-treated (ID) animals showed a significant decline in the PCNA expression, which authenticated the ability of Cu-B to inhibit tumor cell proliferation. Induction of apoptosis in the tissue sections in response to Cu-B was confirmed by the TUNEL assay (**Figures 5E, F**).

The decisive role of the MAPK pathway as observed *in vitro* was validated in the *in vivo* tumor samples. We observed a significant down-regulation in the expression of the key components of the MAPK pathway, p-ERK1/2, and p-MEK1/2, in the lysates of Cu-B-treated mice tissues in comparison to the vehicle control (**Figures 5G, H**). In line with this observation, a strong inhibition in the expression of c-MYC and p-STAT3, a critical downstream target of ERK, supported our *in vitro* data (**Figure 5I**). However, MITF-M, Cyclin-D1, and β-catenin levels were unaltered upon Cu-B treatment (**Figure 5J** and **Figures S4B, C**). The immunohistochemical analysis of the tissue sections authenticated the results obtained from immunoblot analysis (**Figure 5K**). Together, our studies involving a murine model of human melanoma indicate the *in vivo* therapeutic efficacy of Cu-B against melanoma by targeting the MAPK pathway.

## Discussion

Systematic analysis of the bioactive compounds derived from plants of ethnobotanical significance has paved the way for novel lead structures and has largely progressed as drugs against many diseases including cancer (25). The present study was designed to explore the potential anti-cancer activity of *C. epigaeus*, focusing on the rhizome part which is widely being utilized in ethnomedicine. Our study also intended to isolate the principle, if any, which contributes to the anticancer potential of *C. epigaeus* rhizome. Our studies revealed the potent anticancer property of the ethyl acetate extract of *C. epigaeus* rhizome and isolated a cytotoxic fraction, ECF, which yielded Cu-B as the bio-active ingredient. We used cancer cell lines of various tissue origins to screen their sensitivity towards organic extracts of the rhizome, ECF and Cu-B. Amongst the cancer cell lines of various tissue origins, which we employed for the drug screening, the melanoma cell line, A375 was found to be the most sensitive towards ECF and Cu-B. Hence, we tested the cytotoxic potential of Cu-B in melanoma cell lines belonging to two molecular subgroups, based on the difference in their mutation status in RAS and RAF genes. Moreover, in normal skin fibroblast, Cu-B induced 50% cell death only at a concentration three times than in the melanoma cell line.

A literature survey shows that there are up to 40 known cucurbitacins or their derivatives which are essentially classified into 12 groups. Group B cucurbitacins have been shown to possess potent anticancer activity in a variety of cancers *in vitro* and *in vivo* (26). However, this is the preliminary report demonstrating the molecular mechanism underlying the efficacy of Cu-B against human melanoma *in vitro* and *in vivo*. In the melanoma cell line A375, Cu-B induced potent cytotoxicity with an IC50 value of 5 nM. Moreover, analysis of Cu-B induced cytotoxic mechanism in A375 cells showed that the drug potentiates apoptosis involving both the intrinsic and extrinsic pathways. Activation of initiator and executioner caspases, 9 and 3 respectively as well as cell-surface death receptor-mediated caspase 10 and Bid underscore the significant role of mitochondrial pathway in Cu-B induced apoptosis. Our investigation of the underlying reason for the augmented sensitivity of melanoma cells to Cu-B revealed that the drug down-regulates MAPK signaling, involved in cell proliferation.

Melanoma is a subtype of skin cancer, partly driven by the MAPK signaling pathway through RAS-RAF-MEK-ERK signaling, which concludes in the activation of ERK, and regulates p-STAT, MITF-M, c-MYC, and other transcription factors, resulting in alteration of cell proliferation and survival. The most prevalent gene mutations identified in melanoma are B-RAF, RAS, and NF-1, all of which cause constitutive MAPK signaling (20). Evaluation of the cytotoxic potential of Cu-B in melanoma cell lines viz. SK-MEL-2 and SK-MEL-28 with N-RAS and B-RAF mutation respectively revealed that the compound is highly efficacious against the melanoma cell lines, irrespective of the mutation status. In fact, elucidation of the key mutations that drive melanoma progression has resulted in targeted therapies using small-molecule inhibitors of B-RAF and MEK either alone or in combination and has made significant progress in the treatment of melanoma (20, 27). However, acquired resistance poses serious limitations to the success of these small-molecule kinase inhibitors in the clinic (12, 28). A previous study conducted in the human melanoma xenograft model had shown that melanoma cells can transcriptionally up-regulate the B-RAF molecule to compensate for the inhibition by, the B-RAF^V600E^ inhibitor, vemurafenib (29). The present study demonstrates the efficacy of Cu-B, in suppressing the expression of mutant B-RAF^V600E^ protein as well as inhibiting the B-RAF^V600E^ kinase activity as evidenced by inhibition of MEK1/2 phosphorylation. Moreover, Cu-B inhibited both the constitutive as well as EGF-induced ERKphosphorylation, indicating the role of MAPK signaling in regulating the chemotherapeutic potency of Cu-B against melanoma. As RAF inhibitors have been found to relieve the ERK1/2- dependent feedback inhibition of MAPK signaling, inhibition of MEK1/2 along with B-RAF is considered a promising strategy in the treatment of B-RAF -mutated melanoma and MEK inhibition has proved to be beneficial for NRAS-mutated melanoma (30–32). Indeed, molecular docking studies have revealed that cucurbitacins show a significant binding towards the crystal structure of RAF and MEK, comparable to that of the standard B-RAF and MEK inhibitors, imparting cucurbitacins the ability to inhibit the ERK activation in melanoma cells (33). In line with the ability of Cu-B to inhibit MAPK signaling, we also demonstrate the efficacy of Cu-B in down-regulating the downstream effector transcription factors of ERK, such as p-STAT-3 and c-MYC in A375 cells. In melanoma cells harboring B-RAF^V600E^ mutation, MITF-M down-regulated by B-RAF signaling is considered a crucial event for the progression of melanoma (34). However, our study did not find any significant variation in the expression of MITF-M in response to Cu-B at the concentrations investigated. Furthermore, our study using a tumor xenograft model in NOD-SCID mice harboring the B-RAF^V600E^ mutated A375 cells, resulted in substantial inhibition of tumor growth without any apparent toxicity and corroborated our *in vitro* data on the molecular mechanism underlying the anti-melanoma activity of Cu-B. Taken together, the data highlights the potent anti-melanoma activity of Cu-B, involving potentiation of apoptotic cell death and suppression of proliferation by the inhibition of MAPK signaling. Studies are in progress to elucidate the role of Cu-B in modulating key mutations in other genes which cause de-regulated MAPK signaling, viz. RAS and NF-1. Further, the anti-melanoma efficacy of Cu-B has to be evaluated using patient-derived melanoma xenograft model and using patient-derived melanoma cells so that the compound can be effectively translated from bench to bedside.

**Figure 6:**
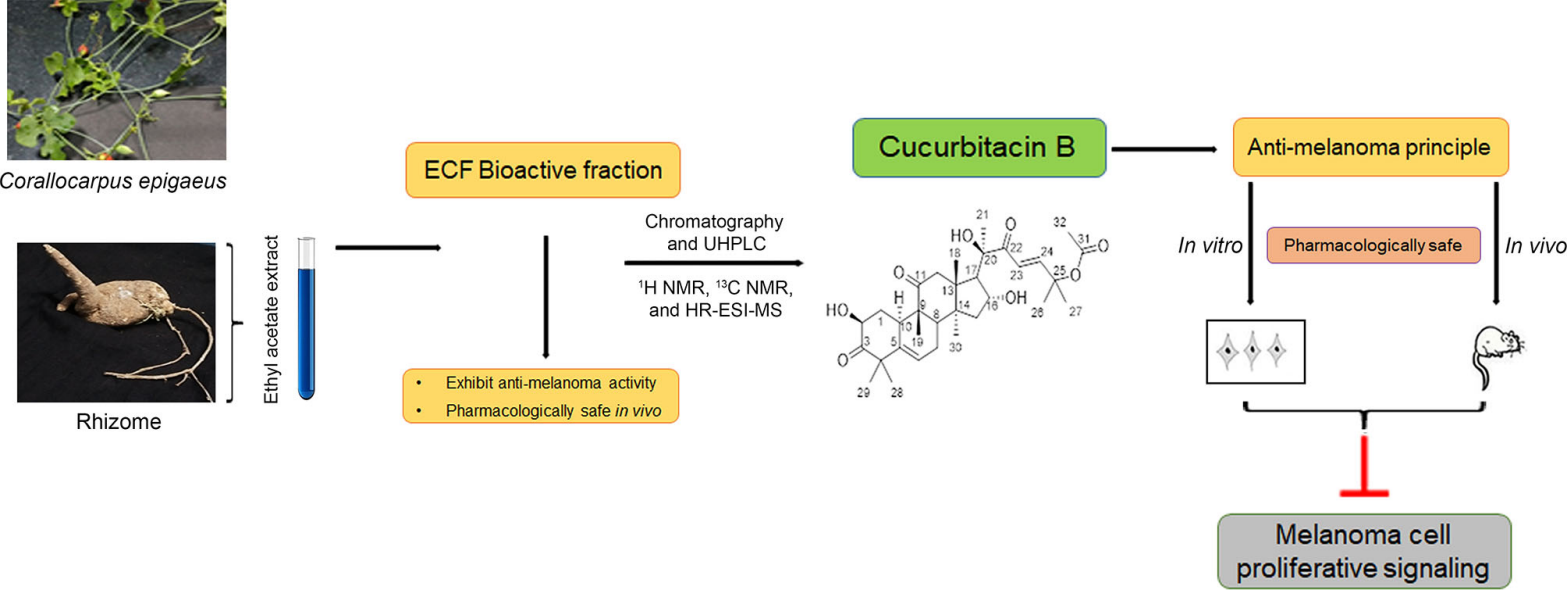
Schematic representation highlighting the isolation process and characterization of the anti-melanoma principle, Cucurbitacin B from the rhizome of C*. epigaeus*. Briefly, ethyl acetate extract of *C. epigaeus* rhizome, was fractionated using column chromatography, which yielded purified Cu-B and the isolated anti-cancer principle was characterized and verified using ^1^H NMR, ^13^C NMR and HR-ESI-MS analysis. *In vitro* and *in vivo* studies validated the potential of Cu-B to attenuate melanoma by targeting critical proliferative signals. The pharmacological safety of ECF and ECF-derived Cu-B were assured in *Swiss albino* mice.

The current study, which explains the derivation of the anti-cancer principle, Cu-B, from *C. epigaeus* and its prospective anti-melanoma efficacy is briefed in a schematic representation (**Figure 6**). To summarize, we report a hidden anti-cancer property displayed by Cu-B, purified from the Cucurbitaceae succulent, *C. epigaeus*, against melanoma. This is the first study reporting the isolation and identification of cucurbitacin B from *C. epigaeus* and also demonstrating its anti-melanoma potential, *in vitro* and *in vivo*. Our study demonstrates the necessity of advancing Cu-B, as a candidate drug against melanoma, which is the most aggressive and treatment-resistant cancer, and accounts for 75% of all skin cancer-related deaths (30).

## Data Availability Statement

The original contributions presented in the study are included in the article/**Supplementary Material**. Further inquiries can be directed to the corresponding authors.

## Ethics Statement

The animal study was reviewed and approved by 326/GO/ReBiBt/S/2001/CPCSEA.

## Author Contributions

Conception and design: RA and SB. Development of methodology: RA, SB and RL. Acquisition of data: AS, GV, LN, SA, MS, TR, and CK. Data editing and Figure arrangement: NH and VL. Isolation Identification and characterization of the compound: RL and MR. Verification of Histopathological data: SS. Review and editing of the manuscript: RA, SB, and NA. All authors contributed to the article and approved the submitted version.

## Funding

This work was supported by the Institutional fund by DBT, Government of India, and extramural grant by KSCSTE Thiruvananthapuram, Kerala to RA (Grant number: 025/SRSLS/2014/CSTE), and extramural grant by KSCSTE Thiruvananthapuram, Kerala to SB (Grant number: 025/SRSLS/2014/CSTE).

## Conflict of Interest

The authors declare that the research was conducted in the absence of any commercial or financial relationships that could be construed as a potential conflict of interest.

## Publisher’s Note

All claims expressed in this article are solely those of the authors and do not necessarily represent those of their affiliated organizations, or those of the publisher, the editors and the reviewers. Any product that may be evaluated in this article, or claim that may be made by its manufacturer, is not guaranteed or endorsed by the publisher.
