# Corrigendum: Cucurbitacin B, purified and characterized from the rhizome of *Corallocarpus epigaeus* exhibits anti-melanoma potential

**DOI:** 10.3389/fonc.2022.989283

**Published:** 2022-08-12

**Authors:** Sreekumar Usha Devi Aiswarya, Gowda Vikas, Nair Hariprasad Haritha, Vijayasteltar Belsamma Liju, Anwar Shabna, Mundanattu Swetha, Tennyson Prakash Rayginia, Chenicheri Kizhakkeveettil Keerthana, Lekshmi Raghu Nath, Mullan Vellandy Reshma, Sankar Sundaram, Nikhil Ponnoor Anto, Ravi Shankar Lankalapalli, Ruby John Anto, Smitha Vadakkeveettil Bava

**Affiliations:** ^1^ Department of Biotechnology, University of Calicut, Malappuram, India; ^2^ Division of Cancer Research, Rajiv Gandhi Centre for Biotechnology, Thiruvananthapuram, India; ^3^ Chemical Sciences and Technology Division, Council for Scientific and Industrial Research (CSIR)-National Institute for Interdisciplinary Science and Technology (CSIR-NIIST), Thiruvananthapuram, India; ^4^ The Shraga Segal Department of Microbiology-Immunology and Genetics, Faculty of Health Sciences, Ben-Gurion University of the Negev, Beer Sheva, Israel; ^5^ Department of Pharmacognosy, Amritha School of Pharmacy, Amritha Vishwa Vidyapeetham, Amrita Institute of Medical Sciences (AIMS) Health Science Campus, Ponekkara P.O, Kochi, India; ^6^ Agro-Processing and Technology Division, Council for Scientific and Industrial Research (CSIR)-National Institute for Interdisciplinary Science and Technology (CSIR-NIIST), Thiruvananthapuram, India; ^7^ Academy of Scientific and Innovative Research (AcSIR), Ghaziabad, India; ^8^ Department of Pathology, Government Medical College, Kottayam, India

**Keywords:** *corallocarpus epigaeus*, cucurbitacin B, melanoma, apoptosis, NMR spectroscopy, mass spectrometry

In the published article, there was an error in **Figure 2** as published. The blot quantification graph of **Figure 2C** was duplicated in place of the graph for **Figure 2D**. The corrected **Figure 2** appears below.

**Figure 2:**
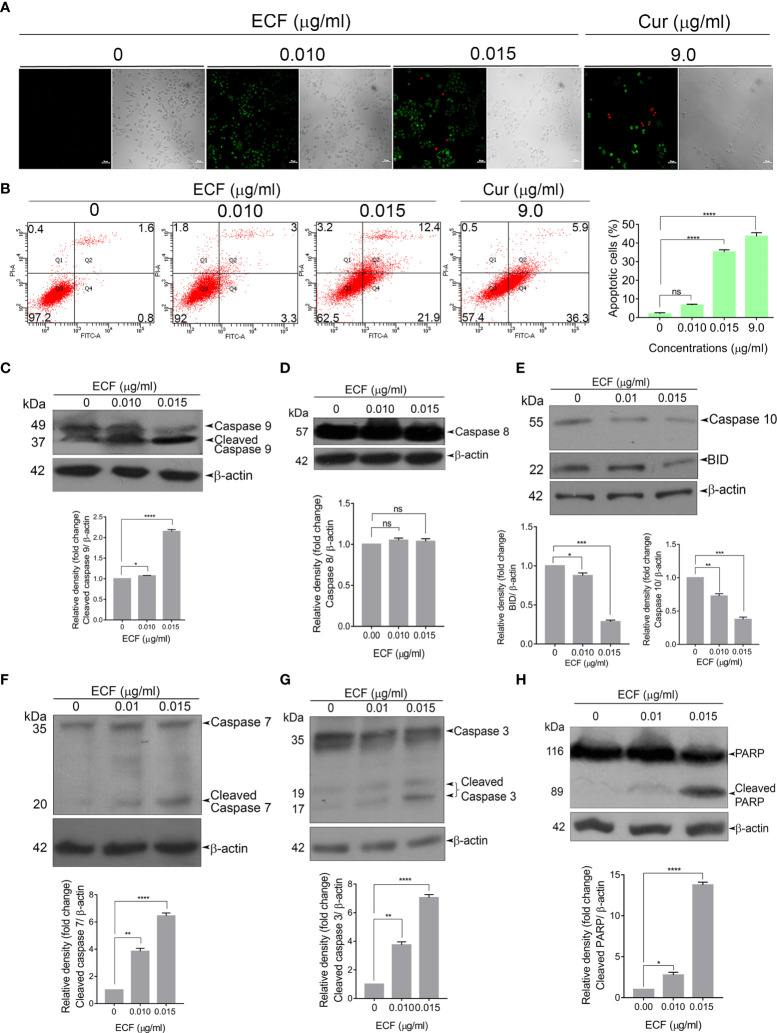
ECF triggers apoptotic mode of cell death in melanoma **(A, B)** ECF induces apoptosis in A375 cells as assessed by Annexin/PI staining, and was quantitated by FACS analysis. **(C–H)** ECF potentiates the activation of caspases and cleavage of PARP in A375 cells as analyzed by immunoblotting. Data are representative of three independent experiments (Mean ± SEM) and P-values are calculated using one-way ANOVA. ****P ≤ 0.0001, ***P ≤ 0.001, **P ≤ 0.01, *P ≤ 0.1and ns ≥ 0.05.

## Publisher’s note

All claims expressed in this article are solely those of the authors and do not necessarily represent those of their affiliated organizations, or those of the publisher, the editors and the reviewers. Any product that may be evaluated in this article, or claim that may be made by its manufacturer, is not guaranteed or endorsed by the publisher.

