# Supplementary material for: Cucurbitacin B, Purified and Characterized From the Rhizome of Corallocarpus epigaeus Exhibits Anti-Melanoma Potential
Source: Front Oncol. 2022 Jun 8;12:903832. doi: 10.3389/fonc.2022.903832 (PMC9213886; doi:10.3389/fonc.2022.903832)
Supplement: Supplementary file 1 [file DataSheet_1.pdf]

Supplementary Materials

Supplementary Figures.

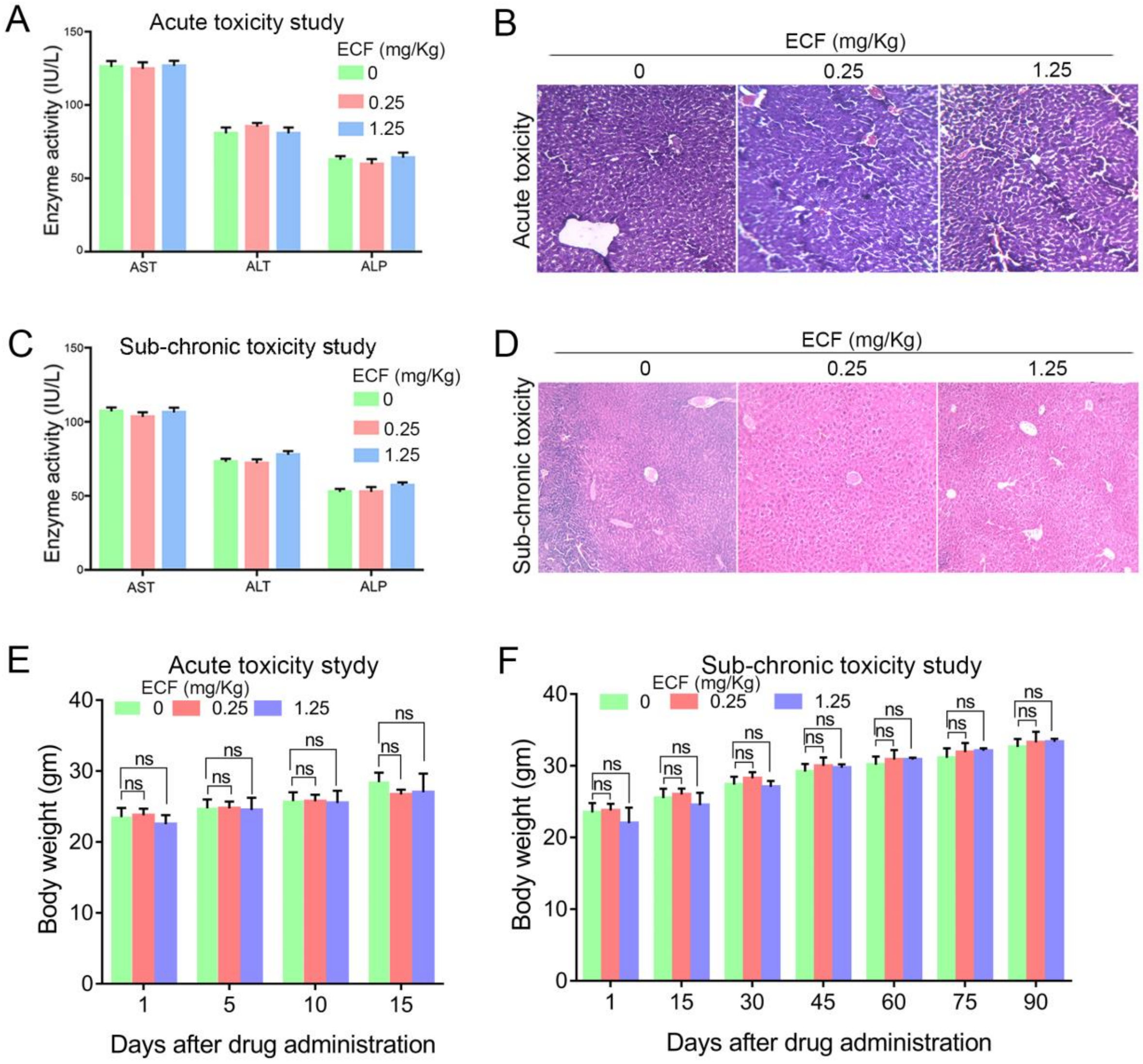

Supplementary Figure 1. Pharmacological safety of ECF, (A-D) Toxicity analysis upon ECF treatment *in vivo.* Graphical representation of AST, ALT, and ALP activities in response to ECF treatment. H&E stained liver tissues of mice treated with ECF. **(E)** Representation of body weight (5 days intervals) of animals of the acute toxicity study. **(F)** Representation of body weight (14 days interval) of animals of the sub-chronic toxicity study.

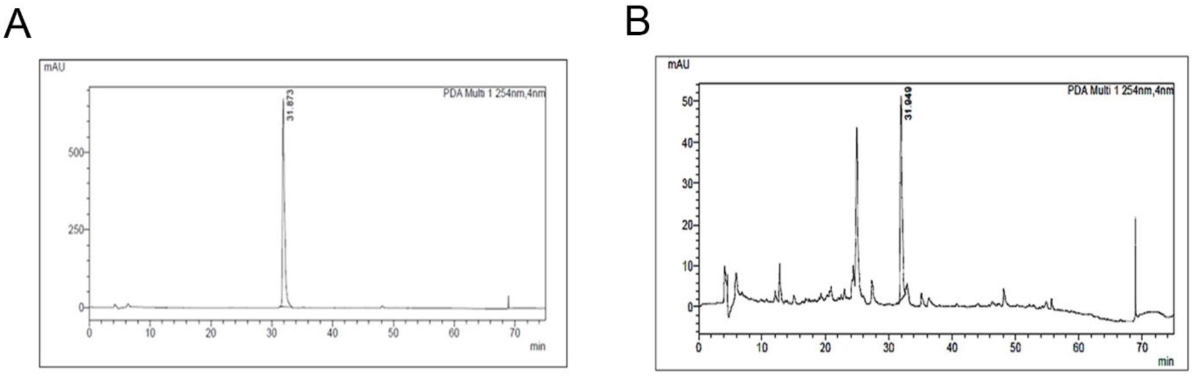

Supplementary Figure 2. UHPLC chromatograms of cucurbitacin B and its presence in ECF fraction, (A-B) ECF fraction (3 mg/ml) and the isolated pure compound (2 mg/ml) were dissolved in acetonitrile:water (1:1) and filtered through a 0.2 µm nylon filter. The sample injection volume was 20 µL, and the C18 column temperature was 35 °C. The mobile phase system consists of water:acetic acid (100:1) (A) and acetonitrile (B). A step gradient program was used for this analysis as follows: 0% B at 0 min to 40% B at 20 min, 40 to 50% at 30 min, 50 to 60% at 40 min, 60 to 80% at 50 min, 80 to 100% at 60 min, then maintaining at 100% B from 60 to 65 min at a flow rate of 1 ml/min, monitored at 254 nm.

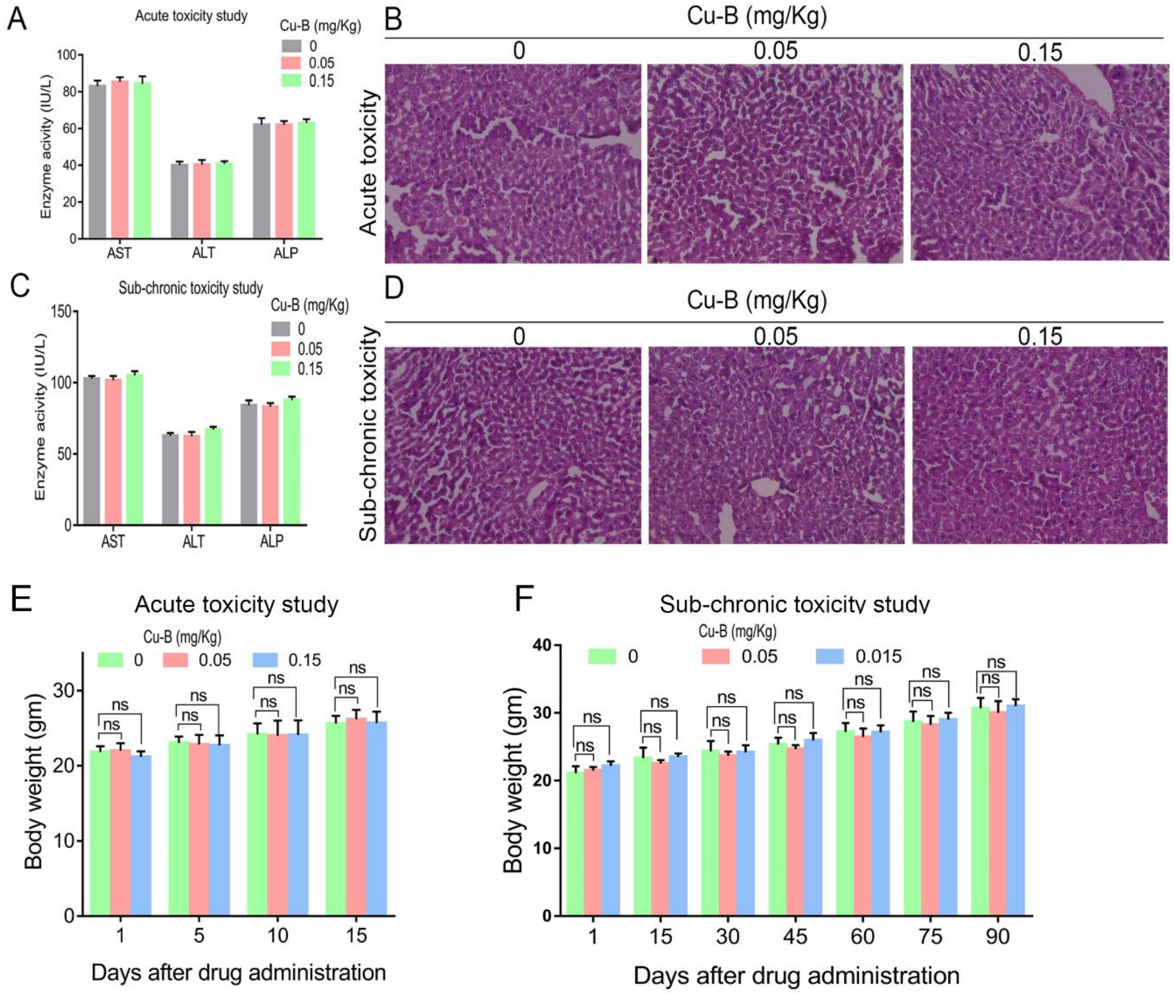

Supplementary Figure 3. Pharmacological safety of Cu-B, (A-D) Acute and sub-chronic toxicity analysis upon Cu-B treatment in *Swiss albino* mice. Graphical representation of AST, ALT, and ALP activities in response to Cu-B treatment. H&E stained liver tissues of mice treated with Cu-B. **(E)** Representation of body weight (5 days intervals) of animals of the acute toxicity study. **(F)** Representation of body weight (14 days interval) of animals of the sub-chronic toxicity study

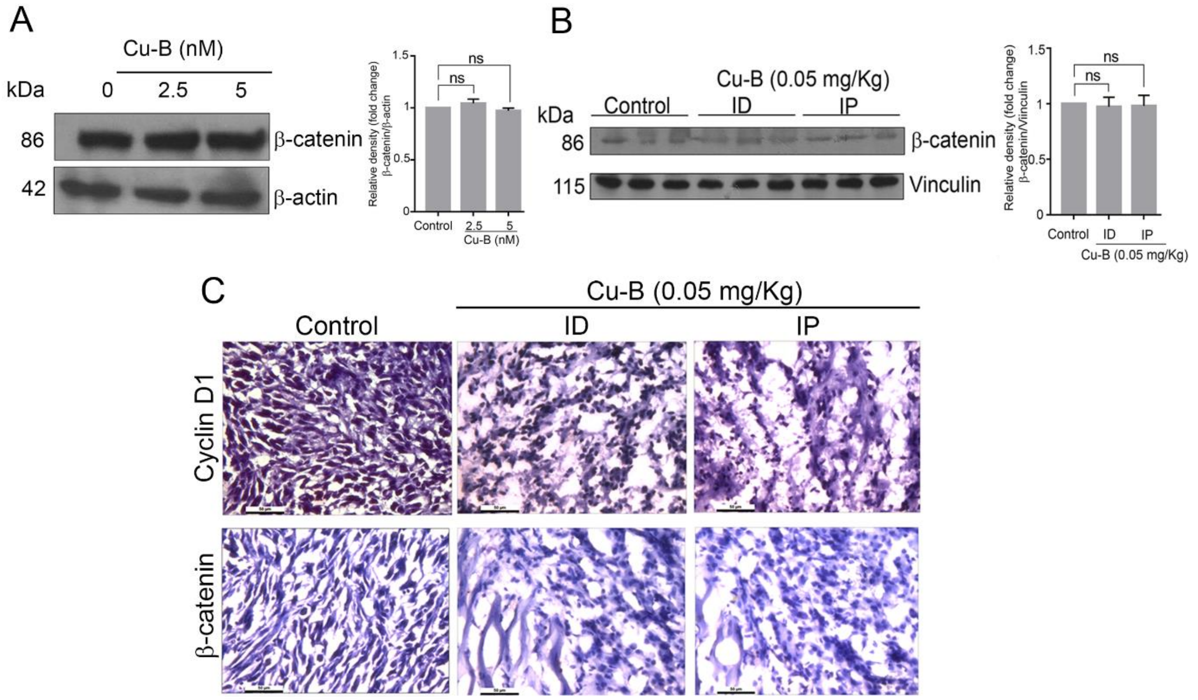

Supplementary Figure 4. The effect of Cu-B on the key survival signals in melanoma, (A) Cu-B treatment unalters β-catenin levels in A375 cells (B) Cu-B does not change β-catenin levels *in vivo* as evidenced in the tumor lysates (C) IHC analysis on the expression of Cyclin D1 and β-catenin in tumor tissues of mice groups. Data are representative of three independent experiments (Mean±SEM) and P-values are calculated using one-way ANOVA. ns ≥ 0.05.

Supplementary Table

Carbon No.	^{1}H (ppm)	^{13}C (ppm)
1	2.30, 1.24	36.01
2	4.36	71.65
3	-	213.08
4	-	50.25
5	-	140.36
6	5.7 (t, *J* = 3.5 Hz, 1H)	120.46
7	1.90 (d, *J* = 7 Hz), 2.42 (d, *J* = 7 Hz)	23.87
8	1.98	42.37
9	-	48.45
10	2.67	33.73
11	-	212.19
12	3.17 (d, *J* = 14.5 Hz), 2.62 (d, *J* = 14.5 Hz)	48.66
13	-	50.69
14	-	48.11
15	1.89, 1.47	45.32
16	4.36	71.3
17	2.5	58.2
18	0.91 (s, 3H)	19.86
19	1.01 (s, 3H)	20.06
20	-	78.24
21	1.35 (s, 3H)	23.93
22	-	202.5
23	6.39 (d, *J* = 16.5 Hz)	120.29
24	7.0 (d, *J* = 16.0 Hz)	152.03
25	-	79.34
26	1.50 (s, 3H)	26.46
27	1.57 (s, 3H)	25.91
28	1.2 4(s, 3H)	29.37
29	1.28 (s, 3H)	21.26
30	1.3 (s, 3H)	18.91
31	-	170.32
32	1.94 (s, 3H)	21.95

Supplementary Table 1. ^{1}H and ^{13}C NMR of cucurbitacin B, in $CDCl_3$, isolated from the ethyl acetate extract of *Corallocarpus epigaeus.*
